# Comparative analysis of volatility forecasting for healthcare stock indices amid public health crises: a study based on the Bayes-CNN model

**DOI:** 10.3389/fpubh.2024.1476196

**Published:** 2024-11-14

**Authors:** Yanguo Li, Ruitao Gu, Dezhi Zhao

**Affiliations:** ^1^School of Economics, Yunnan University of Finance and Economics, Kunming, China; ^2^School of Finance, Yunnan University of Finance and Economics, Kunming, China

**Keywords:** public health crises, healthcare stock indices, volatility forecasting, Bayes-CNN model, comparative analysis

## Abstract

In recent years, public health events have significantly impacted various aspects of human production and daily life, particularly in the domains of disease transmission and economic stability. While many scholars have primarily focused on the influence of public health events from the perspective of disease prevention and control, research examining their economic implications, especially regarding public health indices in the securities market, remains relatively scarce. Such studies are crucial for ensuring public health safety and stability. This paper employs the Bayesian Convolutional Neural Network (Bayes-CNN) model to predict financial market volatility influenced by public health events and conducts a comparative analysis. To validate the feasibility of this method, the model is used to analyze the impact of the COVID-19 pandemic on the CSI (China Securities Index) Medical Service Index. The results indicate significant differences in the volatility of the CSI Medical Service Index before and after the outbreak, particularly during the pandemic period. This study also enhances the validity and reliability of its conclusions by incorporating European data and employing the GARCH model. Relevant institutions and individual investors should adopt different regulatory and investment strategies based on the specifics of various public health events to prevent the outbreak of systemic financial risks that could affect social stability. This paper offers a new perspective and methodology for predicting financial market volatility under the influence of public health events, providing valuable insights for investors and decision-makers to better understand and respond to the potential impacts of such events on financial markets.

## 1 Introduction

Public health events, particularly large-scale infectious disease outbreaks, have increasingly drawn attention to their impacts on the global economy and financial markets. The COVID-19 pandemic, which erupted in 2020, stands as a significant recent public health event that not only caused widespread infections and fatalities in a short period but also triggered a global economic recession and turmoil in financial markets (1). The pandemic has had far-reaching effects on various aspects of the global socio-economic landscape, exposing the vulnerabilities of the global economic system. Market volatility significantly increased during the pandemic, reflecting not only investor uncertainty about future economic prospects but also revealing the market's heightened sensitivity to risk (2). Against this backdrop, the healthcare sector, due to its crucial role in combating the pandemic, became a focal point for market attention. During the stock market crash in the United States triggered by the pandemic, stocks related to the healthcare industry performed exceptionally well (3), indicating that the volatility of healthcare stock indices reflects not only changes in industry supply and demand but also serves as a significant indicator of market sentiment and risk appetite. As the frontline in responding to the pandemic, the volatility of healthcare stock indices was particularly pronounced during this period. Prior to the pandemic, the operation of public health systems was relatively stable, with healthcare resource allocation and utilization rates being consistent; the healthcare sector was viewed as a relatively stable and defensive investment area. However, the outbreak and spread of the virus disrupted this balance and stability, placing immense pressure on public health systems, straining healthcare resources, and impeding socio-economic activities. During the pandemic, healthcare stock indices exhibited significant volatility, influenced by the dynamic developments of the pandemic, policy measures, and public sentiment (4). As the pandemic gradually came under control, healthcare stock indices displayed different patterns of recovery and adjustment in the later stages (5). Post-pandemic, with the gradual relaxation of control measures and the promotion of vaccination, public health systems began to recover, although long-term impacts remain. In such a complex and fluctuating market environment, accurately predicting the trends of healthcare stock indices is of great importance for investors and policymakers. Traditional forecasting methods often struggle to handle the complexity and non-linearity of market data (6). In recent years, advancements in machine learning and deep learning technologies, particularly the application of Bayesian Convolutional Neural Networks (Bayes-CNN), have provided new perspectives and methodologies for research in this field.

Bayesian Convolutional Neural Networks (Bayes-CNN), which combine Bayesian statistics with convolutional neural network technology, have emerged as a promising method in this context (7). Compared to traditional GARCH models and other machine learning models, deep learning models such as CNNs demonstrate higher predictive accuracy and stability when handling financial time series data (8, 9). By incorporating Bayesian methods, Bayes-CNN effectively addresses uncertainty, non-linear relationships, and noise during the modeling process (10). Specifically, Bayes-CNN can adaptively adjust based on historical data and prior knowledge when processing different datasets, thereby enhancing the model's predictive accuracy (11). In contrast to traditional neural networks, Bayes-CNN exhibits greater robustness and generalization capabilities when facing complex and variable uncertainty environments (12). This makes Bayes-CNN a powerful tool for predicting the trends of healthcare stock indices before, during, and after the pandemic. The aim of this study is to conduct a systematic prediction of healthcare stock indices across the pre-pandemic, pandemic, and post-pandemic periods using the Bayes-CNN model. First, this paper will explore the specific impacts of the pandemic on healthcare stock indices and analyze the patterns of their volatility. Next, we will construct the Bayes-CNN model and train it using historical data, evaluating its predictive performance across different time periods. Additionally, to validate the effectiveness and reliability of our conclusions, we will incorporate European data and the GARCH model into the empirical analysis. Finally, this study will discuss the impact of the pandemic on healthcare stock indices based on the predictive results, providing valuable insights for related investment planning and policy formulation.

This study aims to investigate the impact of public health events on the volatility of the CSI Medical Service Index and to conduct a comparative analysis of volatility predictions across three periods: before the pandemic, during the pandemic, and after the pandemic. The research seeks to reveal the specific effects of the pandemic on the volatility of healthcare stock indices. This study holds significant theoretical and practical implications. Theoretically, by comparing the volatility of healthcare stock indices across different time periods, this research enriches the theoretical understanding of the relationship between public health events and financial market volatility. In particular, the application of the deep learning Bayes-CNN model for volatility prediction offers a new method and perspective for predicting financial market volatility (13). Practically, the findings of this study provide valuable insights for investors regarding risk management during public health events, assisting them in making more informed decisions in uncertain market environments. Additionally, policymakers can leverage the findings to better understand the impact of the pandemic on the healthcare industry and financial markets, allowing them to develop more effective market regulation and intervention measures to maintain financial market stability.

The structure of this paper is as follows: the second section is a literature review that examines relevant research on public health events and financial market volatility, volatility forecasting models, and the Bayes-CNN model. The third section introduces the data and methodology of the study, including data sources, model construction, research design, and evaluation metrics. The fourth section presents the empirical analysis, showcasing the optimization results of the models and the predicted volatility across different periods, along with comparative and validity analyses. The fifth section concludes the paper by summarizing the main findings, discussing the limitations of the research, and suggesting directions for future studies.

## 2 Literature review

The impact of public health events on financial markets has gradually become a focal point of research in recent years. Baker et al. (2) demonstrated that the COVID-19 pandemic led to a sharp increase in global economic uncertainty and a significant rise in financial market volatility. Their study quantified the pandemic's impact on financial markets through the analysis of an economic uncertainty index, finding that stock market volatility increased markedly during the pandemic. In the early stages of the outbreak, investors, facing heightened uncertainty, generally shifted toward safe-haven assets, resulting in capital outflows from the stock market and declining prices. Al-Awadhi et al. (14) further analyzed the short-term effects of the pandemic on the Chinese stock market, revealing that the rapid spread and worsening situation of the pandemic led to a significant decline in stock prices across China. In this context, investor sentiment became more cautious, leading to decreased market liquidity and increased market volatility. Wagner and Ramelli (4) studied the pandemic's effects on corporate stock prices, finding that the pandemic increased systemic market risk. Due to supply chain disruptions, reduced demand, and production halts, many companies experienced substantial declines in stock prices in the early stages of the pandemic, resulting in severe market adjustments. However, certain sectors, such as technology and healthcare, performed relatively well due to increased demand and adaptability of their business models. Zhang et al. (1) provided a comprehensive analysis of financial markets, pointing out significant differences in the pandemic's impact across various financial markets. Notably, developed and emerging markets exhibited different responses to the pandemic's shocks. Developed countries, with relatively stable economic foundations, experienced faster recovery in financial markets, while emerging markets faced greater pressure and a slower recovery (15). Goodell (16) emphasized the long-term impacts of public health events on financial markets, noting that pandemics not only have short-term market shocks but may also lead to long-term structural economic changes. Financial markets must adapt to these changes and reassess risks and returns. Additionally, research indicates that public health events can also affect governments and the public. For example, public sector provision of insurance may crowd out private sector initiatives, the public's attitudes toward government may change due to the pandemic, and social trust can influence the financial system. Barro et al. (17) validated this viewpoint through historical data analysis of past public health events, indicating that pandemics similar to COVID-19 typically impact overall economic activity, leading to declines in stock prices and increases in volatility.

Under the influence of public health events, the performance of healthcare stock indices exhibits unique characteristics and patterns of variation. During the COVID-19 pandemic, healthcare stock indices demonstrated significant volatility; however, compared to other sectors, the healthcare industry overall displayed greater stability and growth potential. Due to its crucial role in public health events, the healthcare sector exhibited heightened volatility and trading activity during the pandemic. Mazur et al. (3) indicated that during the COVID-19 pandemic, the performance of the healthcare and technology sectors surpassed that of other industries, with a notable increase in investor demand for these sectors, highlighting the importance of studying the volatility of healthcare stock indices. In the early stages of the pandemic, healthcare stock indices may have experienced short-term declines influenced by market panic. However, as the pandemic spread and demand for healthcare surged, these indices quickly rebounded, displaying a strong upward trend. Al-Awadhi et al. (14) found that during the pandemic, stocks in the information technology and pharmaceutical manufacturing sectors significantly outperformed the market. Expectations for the healthcare sector likely shifted dramatically during the pandemic, and these changes were directly reflected in the performance of healthcare stock indices. As the pandemic progressed, the investment returns in the healthcare sector notably exceeded those of other industries, particularly for companies with core competencies in pandemic prevention and treatment. Pagano et al. (18), using the COVID-19 pandemic as an experiment, concluded that asset markets allocated time-varying prices for companies' disaster risk exposures, revealing how the market prices the resilience of different companies to disaster risks and how this pricing reflects shifts in investor perceptions of risk as disasters unfold. This research can provide insights into the price volatility of companies in the healthcare sector. Given its pivotal role in pandemic control, the healthcare sector attracted substantial capital inflows, becoming a significant choice for investors seeking safe havens and stable returns. Furthermore, Hunjra et al. (19) examined the impact of government health measures during COVID-19 on the volatility of capital markets in East Asian economies, finding that different health policy measures influenced investor behavior and resulted in stock market volatility. Gheorghe et al. (20) studied the relationship between national healthcare system performance and stock volatility during the COVID-19 pandemic, noting that the connection between these two variables was significantly stronger during the pandemic. Su et al. (21) explored the relationship between healthcare financial expenditures (FE) and the pharmaceutical sector stock index (SP), indicating that there exists both positive and negative correlations between FE and SP, which should be examined in conjunction with other events and market conditions. The healthcare stock indices studied in this paper are closely related to government health measures.

Traditional financial market forecasting methods often struggle with complex and nonlinear data. The GARCH model proposed by Bollerslev (22) effectively captures volatility clustering effects in financial time series by considering conditional heteroscedasticity. However, the GARCH model may have limitations when faced with complex nonlinear data. Gunnarsson et al. (23) highlighted that AI and machine learning (ML) methods show promising effectiveness in volatility forecasting, often providing results comparable to or better than those of econometric methods. Lu et al. (24) found that machine learning models outperformed traditional forecasting models in predicting oil futures volatility, indicating that machine learning can effectively handle non-linearities in data sequences and capture important information related to oil futures market volatility. Deep learning models, such as Convolutional Neural Networks (CNN), Recurrent Neural Networks (RNN), and Long Short-Term Memory networks (LSTM), may exhibit superior performance in processing high-dimensional, and non-linear data. Fischer and Krauss (25) investigated the predictive power of LSTM in financial markets, discovering that LSTM networks outperformed memoryless classification methods, such as Random Forest (RF), Deep Neural Networks (DNN), and Logistic Regression (LOG), demonstrating significant advantages in capturing complex patterns in time series data. Bayesian Convolutional Neural Networks (Bayes-CNN) offer a new approach to effectively handle uncertainty and non-linear relationships in volatility forecasting by combining Bayesian statistics with convolutional neural networks. This method captures complex data patterns while quantifying prediction uncertainty. Hernández-Lobato and Adams (26) utilized probabilistic backpropagation in Bayesian neural networks and found it to have higher robustness and predictive accuracy when handling high-dimensional data, along with faster computational speed. Gal and Ghahramani (11) proposed that using dropout as a Bayesian approximation can effectively quantify uncertainty in neural networks without sacrificing computational complexity or testing accuracy. Wei and Chen (27) employed Bayesian Convolutional Neural Networks to quantify uncertainty in solving the inverse scattering problem (ISP), demonstrating the excellent performance of deep learning schemes (DLS). Feng et al. (28) applied Bayes-CNN to predict seismic phase classification, noting that the model can assess uncertainty in predictions and outperforms standard neural networks. These methods can be transferred to financial market predictions to showcase their potential, particularly in addressing high volatility and uncertainty in the market.

Despite the extensive research on the impact of public health events on financial markets and the application of various volatility prediction models, the use of the Bayes-CNN model in economic analysis remains limited, particularly in the context of predicting the volatility of the China CSI Medical Service Index. Specifically, studies employing the Bayes-CNN model to conduct comparative analyses of healthcare stock index volatility across the pre-pandemic, during-pandemic, and post-pandemic phases are still underdeveloped. This paper enriches the literature in this field by systematically analyzing the impact of the pandemic on the volatility of the China CSI Medical Service Index using the advanced Bayes-CNN model, and it provides a comprehensive understanding of the changes in volatility across different phases.

## 3 Data and methodology

### 3.1 Data

This study focuses on the perspective of public health events and selects the CSI (China Securities Index) Medical Service Index as the core indicator of the research, referred to as “CSI Medical Service” with index code 399989. The index is based on a reference date of December 31, 2004, with a base value of 1,000 points. The CSI Medical Service Index includes securities from listed companies in the medical and healthcare industry, encompassing sectors such as medical devices, medical services, and healthcare information technology, thereby comprehensively reflecting the overall performance of healthcare-related companies in China. The CSI Medical Service Index aims to provide investors with an effective market benchmark by integrating the market performance of various sub-sectors within the healthcare industry. The sample stocks of the index cover multiple sub-industries, including medical devices, pharmaceuticals, biotechnology, medical services, and healthcare information technology, effectively showcasing the diversity and overall development trends of the healthcare sector. Specifically, the selection criteria for the CSI Medical Service Index include that companies must be listed on either the main board or the ChiNext board of the Chinese securities market, with their primary business related to the aforementioned healthcare fields. The sample stocks are adjusted annually to ensure the index's representativeness and forward-looking nature. This index not only aids investors in understanding market dynamics within the healthcare sector but also serves as a reference for policymakers regarding industry development. The data involved in this study spans from December 31, 2004, to July 9, 2024, with a daily frequency. Volatility is calculated following the methodology of Zhou and Zhou (29). The specific calculation formula is as follows:


(1) $$\begin{array}{l} vol_{t}=\left(High_{t}-Low_{t}\right)/Avg_{t} \end{array}$$

In Equation (1), *vol*_*t*_ represents the intraday volatility of the CSI Medical Service Index on day *t*. *High*_*t*_ denotes the highest price of the CSI Medical Service Index on day *t*, *Low*_*t*_ denotes the lowest price of the CSI Medical Service Index on day *t*, *Avg*_*t*_ denotes the average price of the CSI Medical Service Index on day *t*. The calculation reflects the maximum volatility of the CSI Medical Service Index on day *t*. Higher volatility indicates more severe fluctuations in financial asset prices, leading to greater uncertainty in asset returns. Conversely, lower volatility signifies more moderate price changes. In this study, volatility is primarily used as a measure of risk. By examining the volatility of the CSI Medical Service Index, we aim to gain a deeper understanding of the impact of public health events on the healthcare industry and to provide valuable insights for investors and policymakers.

### 3.2 Research methodology

#### 3.2.1 Convolutional neural networks

Convolutional Neural Networks (CNNs) are commonly used deep learning architectures for processing image and time series data. The main components of a CNN include convolutional layers, pooling layers, and fully connected layers. The convolutional layer is the core component of a CNN, responsible for extracting local features from the input data through convolution operations. Convolutional layers utilize multiple convolutional kernels (filters) that slide over the input data to generate a set of feature maps. Each convolutional kernel is capable of detecting different features, such as edges, textures, or other patterns. The equation is as follows:


(2) $$\begin{array}{l} \left(f^{*}g\right)\left(i,j\right)=\underset{m}{\sum }\underset{n}{\sum }f\left(m,n\right)\cdot g\left(i-m,j-n\right) \end{array}$$

In this equation, *f* represents the input image, *g* denotes the convolutional kernel, and *i* and *j* are the coordinates of the output feature map. Convolutional layers typically employ non-linear activation functions to introduce non-linearity, further enhancing the model's expressive power (30).

Pooling layers are used to down sample feature maps, reducing computational complexity and the number of parameters while preserving important features. Common pooling methods include max pooling and average pooling. The formula for max pooling is as follow:


(3) $$\begin{array}{l} y\left(i,j\right)=\underset{\left(m,n\right)\in pooling_{region}}{\max}x\left(i+m,j+n\right) \end{array}$$

In this context, *x* represents the input feature map, *y* denotes the pooled feature map, and *m* and *n* are indices representing local regions (the convolutional kernel or pooling area) used to traverse the pixels or feature values within the input image. The pooling layer compresses the feature map by selecting the maximum or average value from the local region, which helps to retain features to some extent while reducing the dimensionality of the data (31). The fully connected layer integrates features from the convolutional and pooling layers and maps them to the output layer. Each node in the fully connected layer is connected to all nodes in the previous layer, similar to traditional artificial neural networks (ANNs). The formula for the fully connected layer is as follows:


(4) $$\begin{array}{l} y=f\left(Wx+b\right) \end{array}$$

In this formula, *W* represents the weight matrix, *x* is the input vector, *b* is the bias vector, *f* is the activation function, and *y* denotes the output of the fully connected layer. The fully connected layer is typically located at the end of the network, mapping high-level abstract features to the final classification or regression results (32).

#### 3.2.2 Bayesian inference

Bayesian Inference introduces Bayesian statistical methods to probabilistically handle the parameters of CNN models, thereby capturing uncertainties in the data. This process mainly involves Variational Inference and Markov Chain Monte Carlo (MCMC) methods. The goal of Bayesian inference is to update the posterior distribution of model parameters by combining prior distributions with observed data (33). Bayes' theorem provides the mathematical foundation for this process, as expressed in the following formula:


(5) $$\begin{array}{l} P\left(\theta |X\right)=\frac{P\left(X|\theta \right)P\left(\theta \right)}{P\left(X\right)} \end{array}$$

In this context, θ represents the model parameters, *X* denotes the observed data, *P*(θ|*X*) is the posterior distribution, *P*(*X*|θ) is the likelihood function, *P*(θ) is the prior distribution, and *P*(*X*) is the marginal likelihood. Bayesian inference reflects the uncertainty in the data through the posterior distribution and provides probabilistic estimates of the model parameters.

Bayesian inference involves Variational Inference, which is a deterministic inference method that approximates complex posterior distributions with a parameterized simpler distribution. This is achieved by optimizing the Variational Lower Bound (ELBO) to approximate the true posterior distribution. The goal is to minimize the Kullback-Leibler (KL) divergence between the true posterior distribution and the approximate distribution. The formula is as follows:


(6) $$\begin{array}{l} KL\left(q\left(\theta \right)||p\left(\theta |X\right)\right)=\int q\left(\theta \right)\log\frac{q\left(\theta \right)}{p\left(\theta |X\right)}d\theta \; \end{array}$$

Here, *q*(θ) represents the approximate distribution, and *p*(θ∣*X*) denotes the posterior distribution. By optimizing the Variational Lower Bound, Variational Inference can effectively approximate the posterior distribution while maintaining high computational efficiency in high-dimensional spaces.

Markov Chain Monte Carlo (MCMC) methods are a class of stochastic sampling techniques used to sample from posterior distributions by constructing a Markov chain. Common MCMC methods include the Metropolis-Hastings algorithm and Hamiltonian Monte Carlo (HMC) algorithm. The principle of the MCMC method is as follows:


(7) $$\begin{array}{l} \pi \left(\theta \right)=\overset{N}{\underset{i=1}{\sum }}\delta \left(\theta -\theta_{i}\right)/N \end{array}$$

In this context, π(θ) represents the sample set from the posterior distribution, while θ_*i*_ denotes the samples drawn from the posterior distribution. MCMC methods can generate samples that approximate the posterior distribution, making them suitable for inference in high-dimensional complex distributions. The application of Bayesian inference in predicting volatility in financial markets involves quantifying the uncertainty of model parameters, thereby enhancing the reliability and robustness of predictions. This is particularly important for risk management and investment decision-making, as it helps investors better understand market risks and make more informed decisions.

#### 3.2.3 Bayesian Convolutional Neural Networks

Bayesian Convolutional Neural Networks (Bayes-CNN or BCNN) enhance the ability of traditional Convolutional Neural Networks (CNN) to handle uncertainty and improve predictive performance by integrating Bayesian inference methods. Traditional CNNs often overlook the uncertainties present in financial time series data, such as volatility predictions, resulting in insufficient robustness and generalization capabilities. In contrast, Bayes-CNN effectively captures the uncertainty in the data by treating model parameters probabilistically, thereby enhancing both the accuracy and stability of predictions.

The structure of the Bayes-CNN model is similar to that of traditional CNNs, but there are significant differences in parameter handling and inference methods. The model structure of Bayes-CNN includes the following key components: Convolutional Layers: These layers are used to extract local features from the input data. In volatility prediction, convolutional layers can effectively capture local patterns and trends in time series data. Pooling Layers: These layers are utilized to reduce the dimensionality of feature maps, thereby decreasing computational complexity while preserving important features. Fully Connected Layers: These layers combine and map the extracted features to generate the final prediction results (34). In Bayes-CNN, the weight parameters of the fully connected layers are treated as random variables that follow a specific probability distribution. Bayesian Inference Module: This module is the key differentiator between Bayes-CNN and traditional CNNs. The Bayesian inference module estimates the posterior distribution of the model parameters using methods such as Variational Inference (VI) or Markov Chain Monte Carlo (MCMC).

In volatility prediction, the Bayes-CNN model can be applied through the following steps: Data Preprocessing: Financial time series data are standardized and normalized, and necessary data augmentation is performed. These steps help to reduce data noise and improve the effectiveness of model training. Model Training: Historical volatility data are used to train the Bayes-CNN model. During the training process, it is essential to optimize the Bayesian inference module to estimate the posterior distribution of the model parameters. Variational inference methods typically approximate the posterior distribution by optimizing the Variational Lower Bound, while MCMC methods sample from the posterior distribution by constructing a Markov chain. Continuous adjustments are made to the model's hyperparameters. Model Prediction: After obtaining the optimal combination of hyperparameters, the trained Bayes-CNN model is utilized to predict future volatility. Through these steps, the Bayes-CNN model effectively leverages the characteristics of financial time series data, enhancing the accuracy and reliability of volatility predictions, and providing robust support for investors and risk managers.

### 3.3 Evaluation metrics

This study divides the volatility of the China CSI Medical Service Index into three time periods for prediction analysis and comparison. The first period is pre-pandemic, spanning from December 31, 2004, to December 31, 2019. The second period covers the pandemic, from January 1, 2020, to December 31, 2022. The third period is post-pandemic, extending from January 1, 2023, to July 9, 2024. In the Bayesian Convolutional Neural Network (Bayes-CNN) model, key evaluation metrics such as Mean Absolute Error (MAE), Mean Squared Error (MSE), and Mean Absolute Percentage Error (MAPE) are employed to assess the model's predictive performance.

Mean Absolute Error (MAE) is the average of the absolute errors between the predicted values and the actual values. The formula is as follows:


(8) $$MAE=\frac{1}{n}\sum_{i=1}^{n}\left|y_{i}-{\hat{y}}_{i}\right|$$

where: *n* is the number of samples, *y*_*i*_ is the actual value of the *i*-th sample, ŷ_*i*_ is the predicted value of the *i*-th sample. MAE measures the average deviation of predicted values from the actual values. Its unit is consistent with that of the original data, making it easy to interpret. MAE is robust to outliers as it considers the absolute value of errors rather than their squares, which means it is less sensitive to extreme values.

The Mean Squared Error (MSE) is the average of the squared differences between predicted values and actual values. The formula is given by:


(9) $$MSE=\frac{1}{n}\sum_{i=1}^{n}{\left(y_{i}-{\hat{y}}_{i}\right)}^{2}$$

where: *n* is the number of samples, *y*_*i*_ is the actual value of the *i*-th sample, ŷ_*i*_ is the predicted value of the *i*-th sample. Due to the squaring of the differences, MSE is more sensitive to large errors. This makes MSE useful for penalizing significant deviations more heavily, thus enforcing a stricter reduction of large errors during model training. MSE is often used as an optimization objective function in many machine learning models, especially in regression problems.

The Mean Absolute Percentage Error (MAPE) is the average of the absolute percentage errors between predicted values and actual values. The formula is given by:


(10) $$MAPE=\frac{1}{n}\sum_{i=1}^{n}\left|\frac{y_{i}-{\hat{y}}_{i}}{y_{i}}\right|\times 100\%$$

In the formula, *n* represents the number of samples, *y*_*i*_ denotes the actual value of the *i*-th sample, and ŷ_*i*_ represents the predicted value of the *i*-th sample. MAPE is a percentage metric, which is applicable to data with different scales, making it convenient for comparing the performance of different datasets or models. MAPE provides a ratio of prediction error relative to the actual values, making it easy to understand and interpret, and thus offering intuitive insights.

In the Bayes-CNN model, these metrics are used to evaluate the model's predictive performance, ensuring its accuracy and stability. MAE: Measures the average prediction error of the model, helping to understand the overall performance of the model on the data. MSE: Penalizes large errors, aiding in the optimization of the model to reduce the occurrence of significant deviations. MAPE: Provides the average level of relative error, making it suitable for scenarios where comparisons between different datasets are necessary. By employing these evaluation metrics, one can comprehensively assess the performance of the Bayes-CNN model in prediction tasks, and make adjustments and optimizations to enhance its predictive accuracy and reliability.

## 4 Experiments and results

### 4.1 Data preprocessing

In the volatility prediction of financial time series data, the Bayes-CNN model combines the efficient feature extraction capabilities of CNNs with the robust uncertainty handling of Bayesian inference, enabling it to provide precise predictions and uncertainty assessments. In this study, we first preprocess the data. Financial time series data are standardized and normalized to ensure the stability and consistency of the input data. We employ min-max normalization to scale the original time series data to the range [0, 1], as shown in the following equation:


(11) $$\begin{array}{l} x_{i}^{,}=\frac{x_{i}-x_{\min}}{x_{\max}-x_{\min}} \end{array}$$

The normalized data point, denoted as $x_{i}^{,}$, is obtained from the original data point *x*_*i*_, with *x*_min_ and *x*_max_ representing the minimum and maximum values in the dataset, respectively. This normalization process mitigates the effects of differing scales, allowing for comparisons and processing of features on a uniform scale. During the model training process, historical volatility data are used to train the Bayes-CNN model. The parameters of the CNN component are optimized, and the Bayesian inference module is refined to estimate the posterior distribution of the model parameters. The trained Bayes-CNN model is then utilized to predict future volatility.

### 4.2 Optimization results at different periods

In the Bayes-CNN model, the Bayesian optimization method is a technique used for hyperparameter tuning that leverages Bayesian inference to predict the optimal values of model parameters. The selection of hyperparameters has a significant impact on model performance in deep learning, and Bayesian optimization provides an efficient approach to search for the best combination of these hyperparameters. The specific steps are as follows: (1) Define the Objective Function: First, define an objective function, typically the validation error or loss function of the model, that you aim to minimize in order to find the optimal hyperparameters. (2) Select Prior Distributions: Choose prior distributions for the hyperparameters. These distributions express initial beliefs about the hyperparameters; for example, they may be uniformly distributed over a specific range. (3) Initial Sampling: Perform initial sampling in the hyperparameter space, evaluate the objective function, and use these points as training data to construct a probabilistic model. (4) Construct the Probabilistic Model: Use the data from the initial sampling to build a probabilistic model of the hyperparameters. This is typically a Gaussian Process, which captures the relationship between the hyperparameters and the objective function. (5) Obtain the Posterior Distribution: Update the prior distribution using Bayes' theorem to obtain the posterior distribution. The posterior distribution reflects beliefs about the hyperparameters based on the observed data. (6) Determine the Optimization Point: Use the probabilistic model to identify the next sampling point that provides the most information. This is often achieved by calculating the acquisition function of the probabilistic model, such as Expected Improvement (EI) or Gaussian Process Upper Confidence Bound (GP-UCB). (7) Iterative Optimization: Evaluate the objective function at the selected optimization point and update the probabilistic model with the new data point. Repeat steps 5 and 6 until a stopping condition is met, such as reaching a predetermined number of iterations or no significant improvement in the objective function. (8) Select the Best Hyperparameters: After all iterations are complete, choose the combination of hyperparameters that minimizes the objective function.

Bayesian optimization is a highly effective method for hyperparameter tuning, particularly for expensive evaluation functions, as it can intelligently select sampling points, thereby reducing the number of required evaluations. Additionally, since the Bayes-CNN model itself possesses the capability of uncertainty estimation, the combination of Bayesian optimization can further enhance the model's generalization ability and robustness.

**Figure 1:**
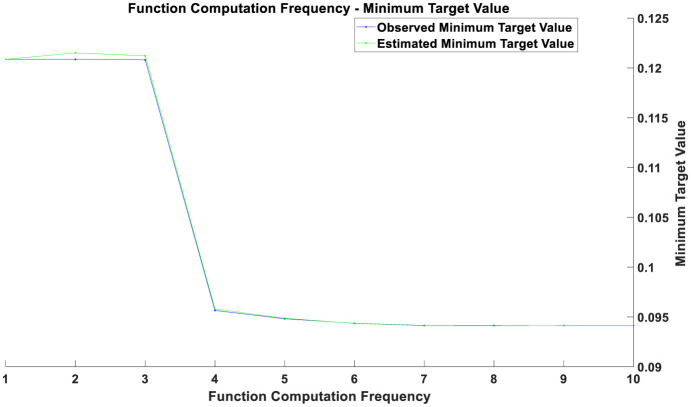
Optimization chart before the pandemic.

**Figure 2:**
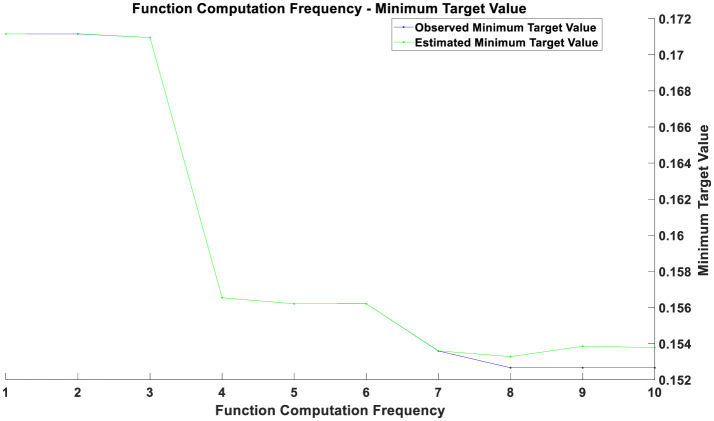
Optimization chart during the pandemic.

**Figure 3:**
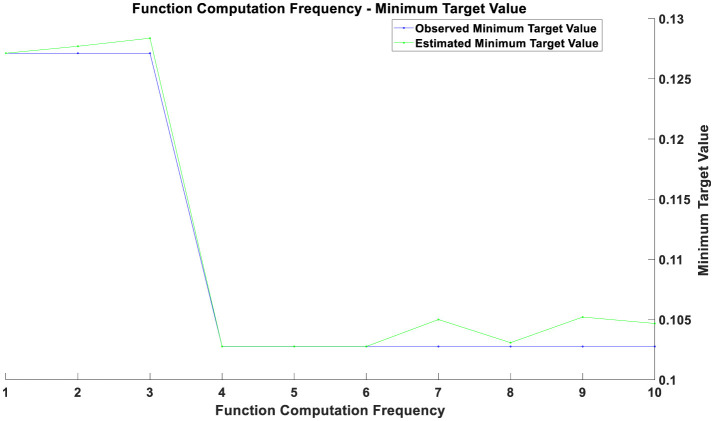
Optimization chart after the pandemic.

Overall, the optimization process consists of two main components. The first component involves optimizing the parameters of the CNN: local features of time series data are extracted through convolutional layers, and the dimensionality of the feature maps is reduced using pooling layers. The Backpropagation algorithm and Gradient Descent optimization are used to adjust the model parameters, minimizing the error on the training data. The second component focuses on the optimization of the Bayesian inference module: this study employs the Markov Chain Monte Carlo (MCMC) method to estimate the posterior distribution of the model parameters. The MCMC method involves constructing a Markov chain to sample from the posterior distribution. The goal of this optimization process is to find the optimal parameter distribution that enhances the model's predictive accuracy and stability when faced with new data. The optimization results before the COVID-19 pandemic are illustrated in Figure 1, those during the pandemic in Figure 2, and post-pandemic optimization in Figure 3. The vertical axis represents the value of the objective function, which is typically minimized in optimization problems. The horizontal axis indicates the number of function evaluations, reflecting the number of iterations or evaluations conducted during the optimization process.

If two curves are close to each other or coincide with each other with a relatively small number of function evaluations, this indicates that the optimization algorithm is efficient and can quickly find solutions near the optimal one. Conversely, if the curves only begin to approach each other after a higher number of function evaluations, it may suggest that the optimization process requires more iterations to converge. During the initial phase (with a smaller number of function evaluations), a rapid decrease in the objective value indicates that the optimization process is quickly approaching the optimal solution. As the number of function evaluations increases, the rate of decrease in the objective value gradually slows down. This may be due to diminishing returns in improvement as the solution approaches the optimal point. The gap between the estimated minimum objective value and the observed minimum objective value decreases over time, reflecting an improvement in the accuracy of the estimated model. From the figures, it can be observed that as the number of function evaluations increases, the observed minimum objective value stabilizes, which may indicate that the optimal solution or convergence point has been approached. The estimated minimum objective value curve closely aligns with the observed minimum objective value curve, suggesting that the estimated model is consistent with the actual observed values.

The optimization results prior to the pandemic, as shown in Figure 1, indicate that the optimization process was continually advancing, with the objective value progressively decreasing. This suggests that the optimization algorithm performed well in searching for the optimal solution. In the initial phase (approximately the first 2–3 training epochs), both training and validation losses were relatively high. This is because the model was just beginning to learn the data features and had not yet been optimized to its best state. Between the 3rd and 4th training epochs, there was a significant decrease in both training and validation losses, indicating that the model rapidly learned the important features of the data during this period, and the optimization process was highly effective at this stage. From the 4th training epoch onward, the training and validation losses stabilized at a low level, suggesting that the model parameters had essentially converged and reached a relatively stable state. Throughout the training process, the training and validation losses were very close and almost coincided, indicating that the model performed consistently across the training and validation sets, with no significant overfitting or under fitting observed. The optimization graph demonstrates that the model converged quickly in the early training stages and maintained stability in the later stages, reflecting good optimization performance on the pre-pandemic data. The significant decrease in the objective value likely indicates that the optimization problem was approaching or had reached a relatively ideal solution.

The optimization results during the pandemic, as depicted in Figure 2, reveal that in the initial phase (approximately the first 3 training epochs), both training and validation losses were high. This is because the model had not yet effectively learned the data features at this early stage. Between the 3rd and 5th training epochs, there was a rapid decrease in both training and validation losses, indicating that the model learned a substantial amount of data features during this period, with significant optimization results. From the 6th training epoch onward, both training and validation losses stabilized at a low level, suggesting that the model parameters had largely converged and reached a stable state. The optimization results post-pandemic, shown in Figure 3, demonstrate that in the initial phase (approximately the first 3 training epochs), both training and validation losses were high, reflecting the model's incomplete learning of data features at this stage. Between the 3rd and 4th training epochs, there was a notable decrease in both training and validation losses, indicating that the model quickly learned the main features of the data, with significant optimization performance. From the 4th training epoch onward, training and validation losses stabilized and remained at a low level, suggesting that the model had largely converged and reached a relatively stable state. After the 4th training epoch, although the training and validation losses remained generally stable, there were minor fluctuations in individual epochs. These fluctuations could be due to some noise or outliers in the data but had minimal overall impact.

By analyzing these optimization plots, we can evaluate the performance of the optimization algorithms across different periods, assess their convergence speed and stability, and gauge the accuracy of the predictive models. Overall, in the training processes across the three periods, the training loss and validation loss are very close and almost overlap. This indicates that the model performs consistently across the training and validation sets, demonstrating good generalization capability with no significant overfitting or underfitting. The model converges quickly in the early training stages and maintains stability in the later stages, reflecting effective optimization performance across different periods. This suggests that the Bayes-CNN model can effectively capture the features of financial time series data from various periods and provide accurate volatility predictions. The model exhibits excellent training and validation performance, with rapid convergence and stability. The introduction of the Bayesian inference module enhances the model's ability to handle uncertainty in the data, providing more reliable predictions. After incorporating Bayesian optimization, the model achieved good optimization with fewer function evaluations. Prior to the pandemic, the optimization process showed rapid improvement and a significant decrease in the objective value. During the pandemic, the optimization process became more stable with smaller fluctuations in the objective value. Post-pandemic, the optimization process demonstrated stability and convergence in the objective value, potentially approaching the optimal solution.

### 4.3 Prediction results across different periods

**Figure 4:**
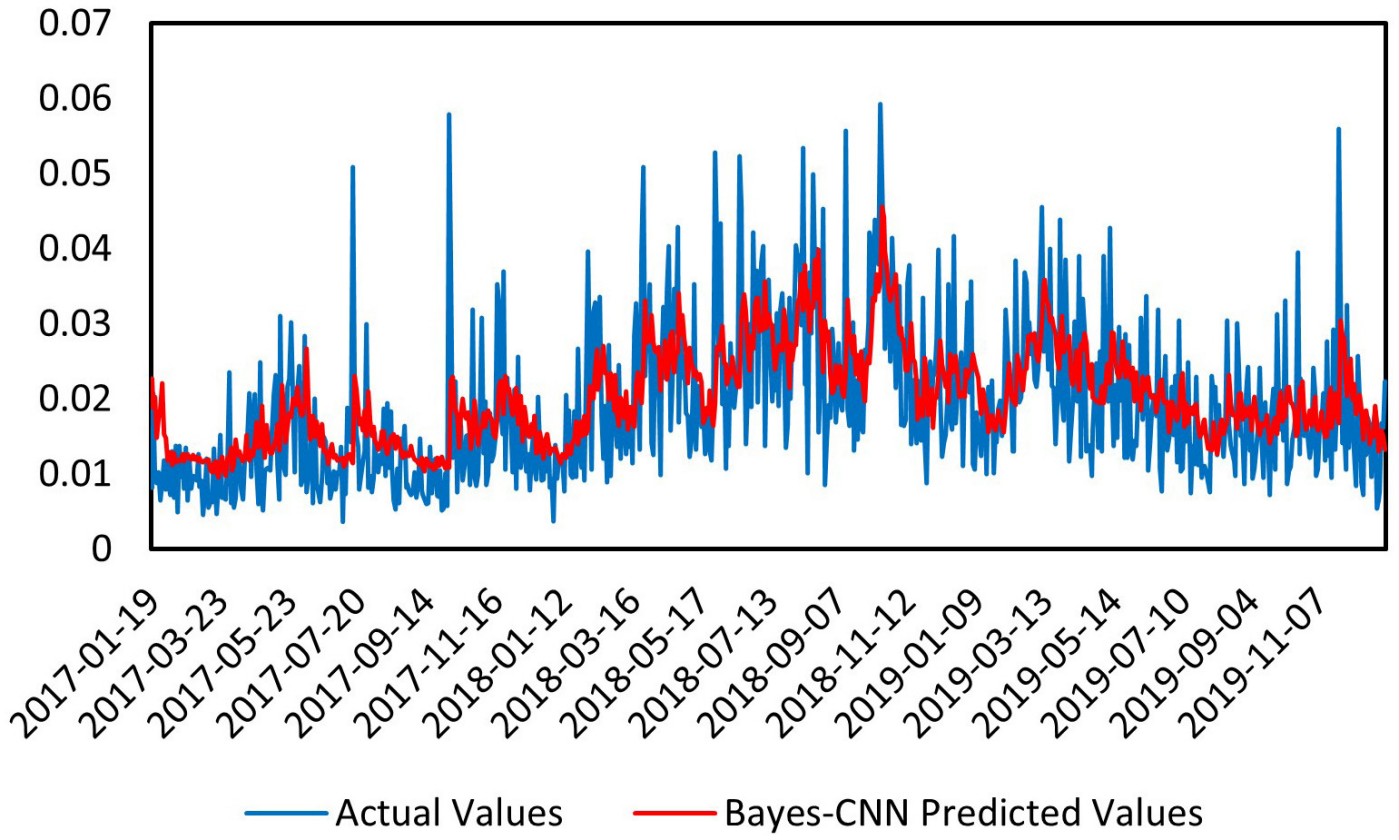
Prediction chart before the pandemic.

First, we conducted a predictive analysis of the volatility of the CSI Medical Service Index for the period prior to the pandemic, from December 31, 2004, to December 31, 2019 . As shown in Figure 4, the vertical axis represents the predicted volatility, while the horizontal axis denotes the time index of the predicted samples. The blue curve reflects the actual volatility, and the red curve illustrates the predicted volatility by the Bayes-CNN model. The overall trend of the predicted values (red) is consistent with that of the actual values (blue), indicating that the model performs well in capturing the overall trend. In areas with significant local fluctuations, the predicted values closely align with the actual values, suggesting that the model also demonstrates good performance in capturing data details. In terms of the various metrics for the first time interval, the Mean Absolute Error (MAE) is 0.006682, which measures the difference between predicted and actual values; a smaller value indicates better predictive performance. The Mean Squared Error (MSE) is 7.6191E-05, another metric for assessing the difference between predicted and actual values, with smaller values reflecting better model performance. The Mean Absolute Percentage Error (MAPE) is 0.4478457, with lower values indicating improved predictive capability. The model effectively fits the overall trend and local details of the data, demonstrating exceptional performance in capturing data features. The Bayesian inference module enables the model to handle uncertainties in the data, further enhancing the reliability of the predictions. Overall, the Bayes-CNN model shows strong predictive effectiveness when processing pre-pandemic data, accurately capturing both the overall trend and local details. The small errors and high prediction accuracy indicate its robustness and reliability. The introduction of the Bayesian inference module allows the model to provide more stable and reliable predictions in the face of data uncertainties.

**Figure 5:**
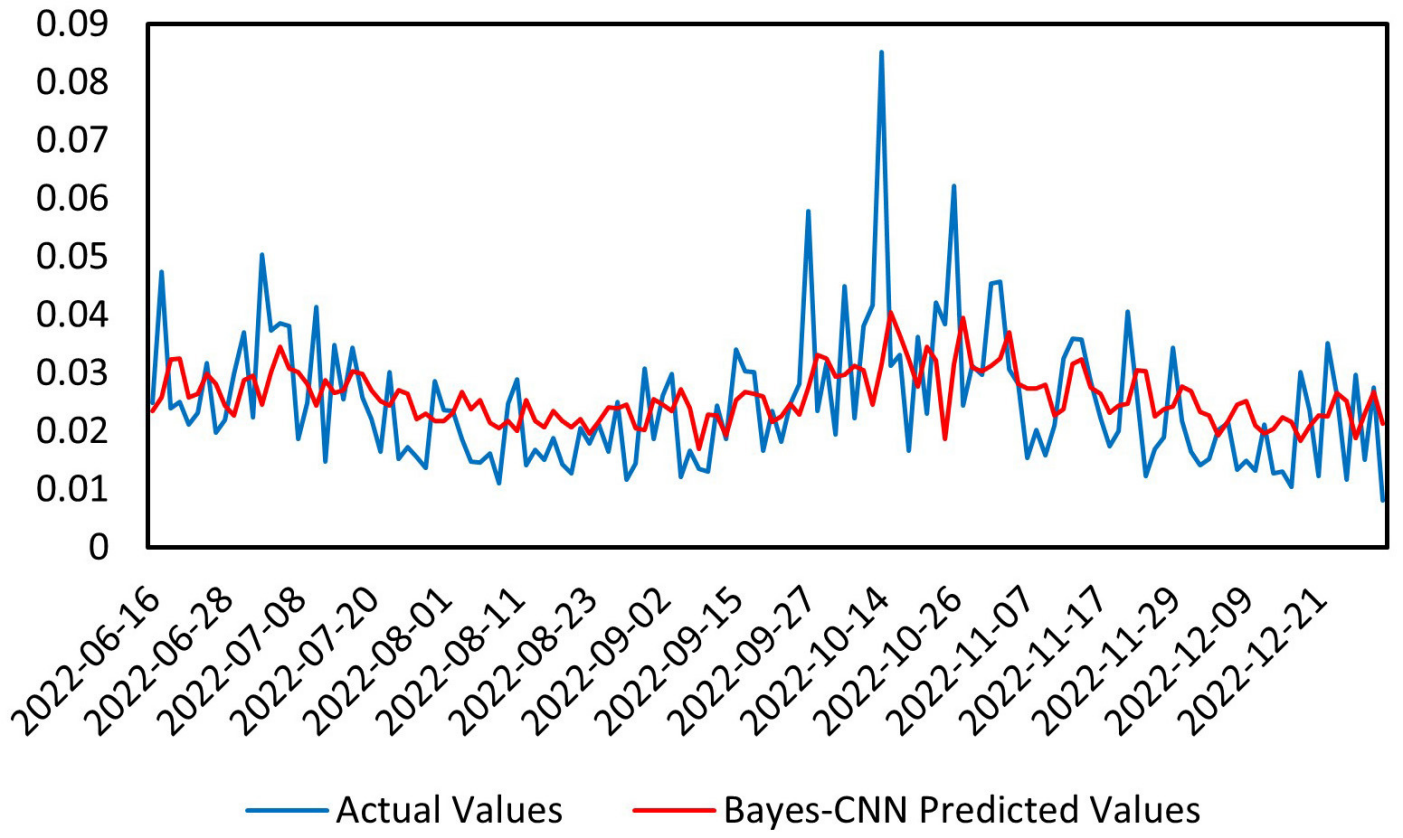
Prediction chart during the pandemic.

Next, we conducted a predictive analysis of the volatility of the CSI Medical Service Index during the pandemic, from January 1, 2020, to December 31, 2022. As illustrated in Figure 5, the overall trend of the predicted values (red) is consistent with that of the actual values (blue), particularly in relatively stable regions, where the model can closely follow the changes in the actual values. However, in areas with significant local fluctuations, there are some discrepancies between the predicted and actual values. This may be attributed to the heightened volatility in the market during the pandemic, which introduced more noise and outliers into the data, complicating the predictions. For the various metrics in this second time interval, the Mean Absolute Error (MAE) is 0.008051, indicating the difference between the predicted and actual values; a smaller value reflects better model performance. The Mean Squared Error (MSE) is 1.1147E-04, another metric for measuring the difference between predicted and actual values, with smaller values indicating superior predictive capability. The Mean Absolute Percentage Error (MAPE) is 0.3726628, where lower values also suggest improved model performance. While the model effectively captures the overall trend of the data, certain discrepancies arise in regions of higher volatility. The MAE and MSE indicate that the prediction errors are within an acceptable range, although they are slightly higher than those observed in pre-pandemic predictions, suggesting that the increased market volatility during the pandemic posed greater challenges to forecasting. The Bayesian inference module enables the model to handle uncertainties in the data, further enhancing the reliability of the predictions. Overall, the Bayes-CNN model demonstrates a good ability to capture the overall trend in the data during the pandemic; however, discrepancies exist between the predicted and actual values in more volatile regions. The slightly higher errors compared to pre-pandemic predictions indicate that the increased market volatility during the pandemic presented additional challenges for the model's forecasts.

**Figure 6:**
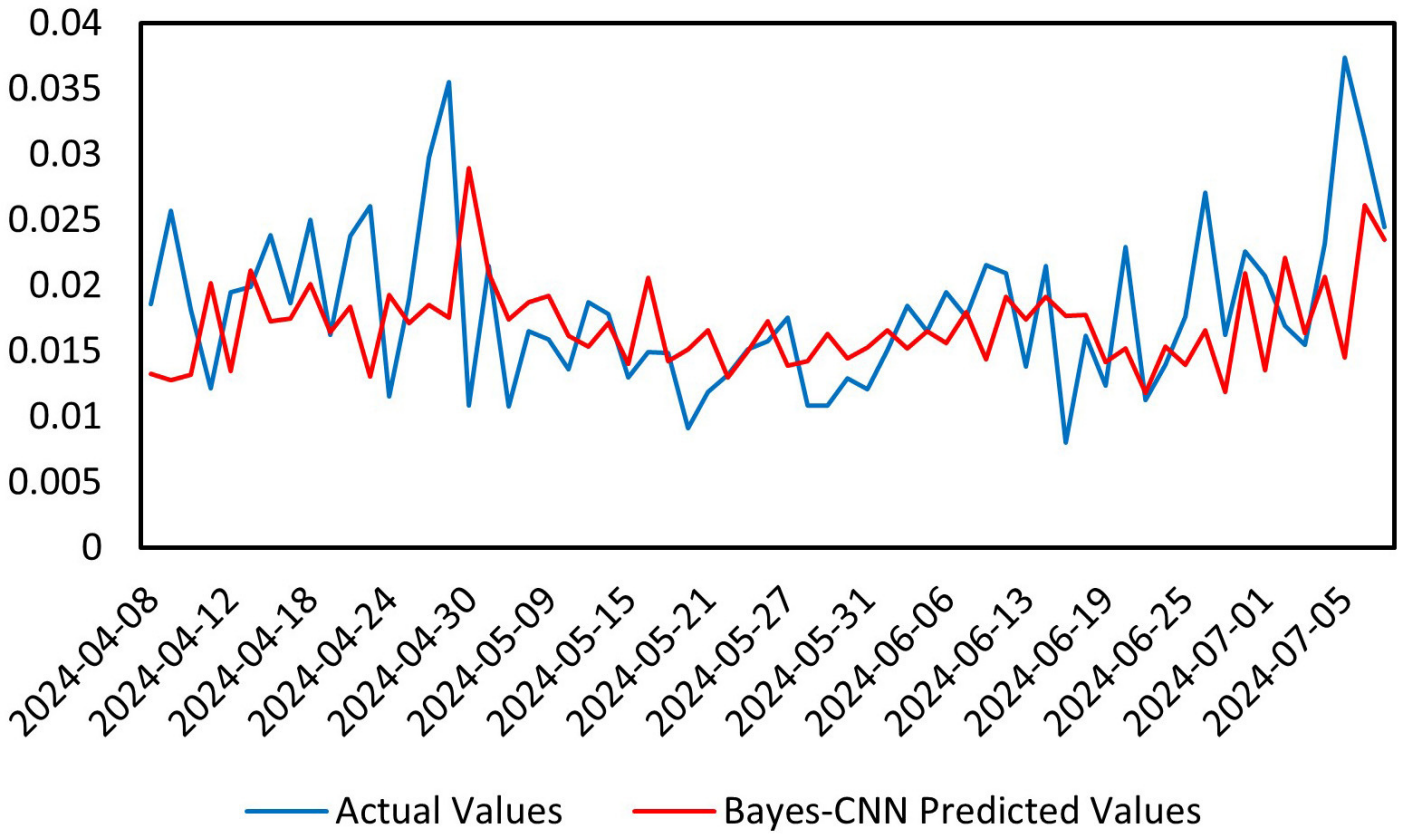
Prediction chart after the pandemic.

Finally, we conducted a predictive analysis of the volatility of the CSI Medical Service Index after the pandemic, from January 1, 2023, to July 9, 2024. As shown in Figure 6, the overall trend of the predicted values (red) is consistent with that of the actual values (blue), particularly in relatively stable regions, where the model effectively follows the changes in the actual values. In areas with significant local fluctuations, the predicted values still maintain a high level of consistency with the actual values, although there are some discrepancies at individual peak points. For the various metrics in this third time interval, the Mean Absolute Error (MAE) is 0.004671, which measures the difference between predicted and actual values; a smaller value indicates better model performance. The Mean Squared Error (MSE) is 4.2973E-05, another metric that assesses the difference between predicted and actual values, with smaller values reflecting superior predictive capability. The Mean Absolute Percentage Error (MAPE) is 0.2691805, where lower values also suggest improved model performance. The model effectively captures both the overall trend and local details of the data, demonstrating outstanding performance in identifying data features. The Bayesian inference module enables the model to handle uncertainties in the data, further enhancing the reliability of the predictions. Overall, the Bayes-CNN model exhibits a strong ability to capture both the overall trend and local details in the post-pandemic data, indicating high predictive accuracy. The small errors and high prediction accuracy suggest that the model possesses strong robustness and reliability.

### 4.4 Comparison summary

A comparison of the primary evaluation metrics for the Bayes-CNN model across the periods before, during, and after the pandemic is presented in Table 1. The metrics include Mean Absolute Error (MAE), Mean Squared Error (MSE), and Mean Absolute Percentage Error (MAPE). These metrics provide insights into the model's predictive accuracy and robustness across different market conditions.

**Table 1:** CSI Medical Service Index Evaluation Metrics Comparison.

**Period**	**MAE**	**MSE**	**MAPE**
Pre-pandemic	0.006682	7.6191E-05	0.4478457
Pandemic	0.008051	1.1147E-04	0.3726628
Post-pandemic	0.004671	4.2973E-05	0.2691805

Pre-Pandemic: The MAE value is 0.006682, indicating that the model's average absolute error was relatively small before the pandemic, reflecting good predictive performance. During the Pandemic: The MAE increased to 0.008051, suggesting that the model's prediction errors grew during the pandemic. This may be attributed to heightened market volatility and increased data complexity during this period. Post-Pandemic: The MAE decreased to 0.004671, indicating a reduction in prediction errors after the pandemic. This suggests that the market may have stabilized, thereby enhancing the model's predictive performance. Pre-Pandemic: The MSE value was 7.6191E-05, signifying that the model had a low mean squared error prior to the pandemic, reflecting high predictive accuracy. During the Pandemic: The MSE rose to 1.1147E-04, indicating a significant increase in prediction errors during the pandemic. This could be due to the intense market fluctuations, which made it challenging for the model to accurately capture rapidly changing trends. Post-Pandemic: The MSE decreased to 4.2973E-05, demonstrating a notable reduction in prediction errors following the pandemic, as the market returned to stability and the model's predictive performance improved significantly. Pre-Pandemic: The MAPE value was 0.4478457, indicating that the model's average relative error was relatively large but still within an acceptable range. During the Pandemic: The MAPE decreased to 0.3726628, reflecting a reduction in the model's relative error during the pandemic. This may be due to changes in the measurement standards for relative error in a highly volatile market. Post-Pandemic: The MAPE further declined to 0.2691805, indicating a significant reduction in the model's relative prediction error under stable market conditions after the pandemic, resulting in notable improvements in predictive performance.

Pre-Pandemic: Before the outbreak of the pandemic, the healthcare industry experienced relative market stability. During this period, the market was primarily influenced by routine healthcare demand, policy changes, and the financial performance of companies. The MAE and MSE of the Bayesian Convolutional Neural Network (Bayes-CNN) were relatively low, indicating good predictive performance of the model in a stable market. However, the higher MAPE suggests that the model had some shortcomings in handling relative errors. During the Pandemic: The outbreak of COVID-19 led to a dramatic increase in demand within the healthcare sector, particularly for medical supplies, vaccines, and testing equipment. The market experienced heightened volatility during this time, and the MAE and MSE of the Bayes-CNN significantly increased, reflecting a rise in prediction errors within a high-volatility market. However, the MAPE showed a slight decrease, possibly due to the adaptability of relative error measurements in a rapidly fluctuating market. As the pandemic was brought under control, the market gradually returned to normal, with the healthcare industry stabilizing. Widespread vaccination and effective control measures contributed to a more stable market sentiment. Post-pandemic, the model's MAE, MSE, and MAPE all showed significant reductions, indicating improved predictive performance in a stable market environment.

In summary, the analysis of the Bayesian Convolutional Neural Network's (Bayes-CNN) predictive performance under different market conditions reveals significant variations in model efficacy. Prior to the pandemic, the market was relatively stable, with moderate error metrics; however, the relatively high MAPE indicated some predictions had substantial percentage deviations. During the pandemic, the market experienced extreme instability, with all error metrics increasing, reflecting the increased difficulty of predictions under such volatile conditions. Post-pandemic, as the market gradually stabilized, the error metrics significantly improved, showcasing the model's best performance with the smallest prediction errors and highest accuracy. This suggests that while the model still requires improvements for handling highly volatile data, it exhibits robust predictive capabilities under stable market conditions.

### 4.5 Validity analysis

To comprehensively assess and validate the reliability, effectiveness, and broad applicability of the conclusions drawn from this study, we implemented two rigorous validation strategies. First, we conducted an extensibility analysis by incorporating a dataset from Europe. This initiative aims to examine the generalizability of our findings across different regional contexts, particularly given that Europe was the second-largest area affected by the outbreak and spread of COVID-19. For this purpose, we selected the STOXX Europe 600 Health Care Index as our European dataset. Second, we introduced the Generalized Auto Regressive Conditional Heteroskedasticity (GARCH) model for predictive analysis. The GARCH model is widely used in the analysis of financial time series data and is effective in capturing the dynamic changes in volatility within financial markets. This helps us more accurately evaluate the potential interference of market fluctuations on our research outcomes. By incorporating this model, we not only validate the stability of our original conclusions in a complex volatility environment but also further elucidate the potential relationships between market volatility and the study subject. This ultimately enhances the persuasiveness and practicality of our research findings.

We divided the volatility of the STOXX Europe 600 Health Care Index into three time periods: before, during, and after the COVID-19 pandemic. Subsequently, we employed the Bayes-CNN model to predict the volatility of the STOXX Europe 600 Health Care Index for each of these three periods. The performance evaluation metrics for the Bayes-CNN model's predictions of the volatility of the STOXX Europe 600 Health Care Index in Europe are presented in Table 2.

**Table 2:** Europe data evaluation metrics comparison.

**Period**	**MAE**	**MSE**	**MAPE**
Pre-pandemic	0.003219	1.8407E-05	0.3709517
Pandemic	0.004169	2.6429E-05	0.3473597
Post-pandemic	0.002887	1.1348E-05	0.4007299

From Table 2, it is evident that in the comparison of MAE and MSE values, the volatility during the pandemic is the highest, followed by the pre-pandemic volatility, while the post-pandemic volatility is the lowest. This indicates that when using the Bayes-CNN model to predict the volatility of the STOXX Europe 600 Health Care Index, the predictions are most accurate post-pandemic, moderately accurate pre-pandemic, and least accurate during the pandemic. This finding is consistent with our predictions for the volatility of the CSI Medical Service Index. However, the results differ when assessed from the perspective of MAPE. This discrepancy may arise from the following four reasons: (1) Differences in Market Environment and Policy Responses: The Chinese government implemented swift and robust control measures during the pandemic, leading to a relatively rapid economic recovery. This may have resulted in reduced market volatility post-pandemic, thus enhancing the model's predictive performance. In contrast, the STOXX Europe 600 Health Care Index was influenced by a combination of policies, economic environments, healthcare resources, and global dynamics in the healthcare industry across multiple European countries. The significant variations in pandemic control measures and economic stimulus policies among European nations led to differing performances in market volatility at various stages. (2) Impact of Economic and Industry Factors: The economic structures and industry characteristics of China and Europe differ, which may affect the performance of the healthcare sector in each region. During the pandemic, the Chinese healthcare industry received more investment and support, whereas the European healthcare sector faced greater challenges, such as unequal distribution of healthcare resources and varying rates of vaccination. (3) Market Sentiment and Investor Behavior: Market sentiment reactions may differ across regions. In China, post-pandemic market sentiment may be more optimistic, with increased investor confidence leading to reduced volatility. Conversely, market sentiment in Europe may exhibit varying characteristics at different stages, particularly during the mid and post-pandemic periods, where it may have been more unstable. Additionally, investor behavior and expectations could also influence market volatility. In China, heightened confidence in economic recovery may have contributed to decreased market volatility, whereas in Europe, investor behavior during various stages of the pandemic could reflect more complex volatility changes. (4) Sensitivity of Evaluation Metrics: The sensitivity and measurement methods of MAE, MSE, and MAPE vary. For the STOXX Europe 600 Health Care Index, the lower MAPE during the pandemic may result from the inherently low volatility during that period. Although predictions exhibit bias, the relative error is not significant. Conversely, as volatility increased post-pandemic, the relative errors also magnified.

In summary, despite some subtle differences, the results obtained from using the Bayes-CNN model to predict the volatility of the CSI Medical Service Index and the STOXX Europe 600 Health Care Index across the pre-pandemic, during-pandemic, and post-pandemic periods are generally consistent. Next, we employed the classic GARCH (1,1) model (35, 36) to conduct predictive analysis on the same dataset and time divisions for the China region. The performance evaluation metrics for these predictions are presented in Table 3.

**Table 3:** GARCH evaluation metrics comparison.

**Period**	**MAE**	**MSE**	**MAPE**
Pre-pandemic	0.006941	7.9477E-05	0.4892502
Pandemic	0.008069	1.0712E-04	0.3752078
Post-pandemic	0.004267	3.2104E-05	0.2656612

It can be observed that the three performance evaluation metrics of the GARCH model are consistent with the results of the Bayes-CNN model: the predictions post-pandemic are the best, followed by those pre-pandemic, while predictions during the pandemic are the least effective. Furthermore, the predictive performance of the Bayes-CNN model is slightly superior to that of the GARCH model both pre-pandemic and during the pandemic. This further validates the conclusions drawn in this study regarding the analysis of markets related to the healthcare sector. The findings obtained through these two thorough and systematic analytical approaches provide strong evidence supporting the reliability, effectiveness, and broad applicability of this research.

## 5 Conclusion

This study aims to investigate the impact of public health events on the volatility of the CSI Medical Service Index and to conduct a comparative analysis of volatility predictions across three distinct periods: before the pandemic, during the pandemic, and after the pandemic. We employed the Bayes-CNN model, a novel predictive model that integrates Bayesian methods with Convolutional Neural Networks to enhance the accuracy and stability of volatility predictions. Initially, we performed detailed data preprocessing on the CSI Medical Service Index, utilizing standardization techniques to ensure the stability and consistency of the data inputs. During the model training process, we trained the Bayes-CNN model using historical volatility data and employed the Markov Chain Monte Carlo (MCMC) method to determine the optimal combination of hyperparameters. Finally, we utilized the trained Bayes-CNN model to predict future volatility and conducted a validity analysis. By comparing the prediction results from the Bayes-CNN model with those from the European dataset and the GARCH model, we further corroborated the findings of this study. Overall, the Bayes-CNN model demonstrated strong predictive capabilities and robustness when handling data across different market conditions. Notably, post-pandemic, as the market began to recover and stabilize, the model's predictive performance improved significantly. This indicates that the Bayes-CNN model possesses high predictive accuracy and reliability under stable market conditions. During the pandemic, despite the increased market volatility, the model was still able to capture the overall trends in the data, although discrepancies were observed in regions with significant local fluctuations.

This study enriches the theoretical research on the relationship between public health events and financial market volatility by conducting a comparative analysis of the volatility of the CSI Medical Service Index across different time periods. It reflects the dynamic changes in markets related to the Chinese healthcare industry. The use of the Bayes-CNN model for volatility prediction introduces a new method and perspective for forecasting financial market volatility. The results offer valuable insights for investors regarding risk management during public health events, aiding them in making more informed decisions in uncertain market environments. Additionally, policymakers can leverage the findings of this study to formulate more effective market regulation and intervention measures to maintain financial market stability.

Future research could focus on further optimizing the Bayes-CNN model, particularly its performance in high-volatility and extreme market conditions. Moreover, integrating other deep learning and machine learning methods into the model could enhance prediction accuracy and stability. Expanding the research to other industries and markets would help validate the model's broad applicability and robustness. These efforts will improve our understanding and response to the impact of public health events on financial markets, providing more reliable and effective support for investors and policymakers.

## Data Availability

The original contributions presented in the study are included in the article/supplementary material, further inquiries can be directed to the corresponding author.
